# The patient perspective in health care networks

**DOI:** 10.1186/s12910-018-0298-x

**Published:** 2018-06-05

**Authors:** Kasper Raus, Eric Mortier, Kristof Eeckloo

**Affiliations:** 1 Ghent University Hospital, Corneel Heymanslaan 10, Ghent, Belgium; 2 Faculty of Arts and Philosophy, Department of Philosophy and Moral Sciences, Ghent University, Blandijnberg 2, Ghent, Belgium; 3 Faculty of Medicine and Health Sciences, Ghent University, Corneel Heymanslaan 10, Ghent, Belgium; 4 Faculty of Medicine and Health Sciences, Department of Public Health, Ghent University, Corneel Heymanslaan 10, Ghent, Belgium

**Keywords:** Health care networks, Health care organization, Ethical duties, Patient perspective, Normative framework

## Abstract

**Background:**

Health care organization is entering a new age. Focus is increasingly shifting from individual health care institutions to interorganizational collaboration and health care networks. Much hope is set on such networks which have been argued to improve economic efficiency and quality of care. However, this does not automatically mean they are always ethically justified. A relevant question that remains is what ethical obligations or duties one can ascribe to these networks especially because networks involve many risks. Due to their often amorphous and complex structure, collective responsibility and accountability may increase while individual responsibility goes down.

**Main body:**

We argue that a business ethics approach to ethical obligations for health care networks, is problematic and we propose to opt for a patient perspective. Using the classic four principles of biomedical ethics (justice, nonmaleficence, beneficence and autonomy) it is possible to identify specific ethical duties. Based on the principle of justice, health care networks have an ethical duty to provide just and fair access for all patients and to be transparent to patients about how access is regulated. The principle of nonmaleficence implies an obligation to guarantee patient safety, whereas the principle of beneficence implies an obligation for health care networks to guarantee continuity of care in all its dimensions. Finally, the principle of autonomy is translated into a specific obligation to promote and respect patient choice. Networks that fail to meet any of these conditions are suspect and cannot be justified ethically.

**Conclusions:**

Faced with daunting challenges, the health care system is changing rapidly. Currently many hopes ride on integrated care and broad health care networks. Such networks are the topic of empirical debate, but more attention should be given to the ethical aspects. Health care networks raise new and pressing ethical issues and we are in need of a framework for assessing how and when such networks are justified.

## Background

Health care organization is entering a new age. Whereas traditionally, individual health care institutions formed the cornerstone of medical care provision, focus is increasingly shifting towards interorganizational collaboration, integrated care, and health care networks (HCNs) [1, 2]. These changes are caused by many factors, including an ageing population and an increasing cost of the welfare system, resulting in medical resources becoming scarcer. Hopes are high for these collaborations and health care networks, which, as studies suggest, could increase economic efficiency [3] and lead to improved quality of medical care [4].

However, the mere fact that HCNs may be beneficial *in particular respects and particular contexts* does not automatically mean they are *always* ethically justified. Economic efficiency, for example, can come at an ethical cost. A market-driven HCN could well be perfectly economically efficient but ethically unjust in failing to provide basic care for those worse off. A 2005 study showed that for-profit hospitals are less likely than not-for-profit hospitals to offer care that promises less potential profit for the institutions [5]. This means it is possible that a particular for-profit hospital or HCN might fail to offer basic care to patients on the grounds that it is not profitable, or they might offer it at an unfairly high price to guarantee its profitability.

Likewise, increasing quality of care does not guarantee ethical justifiability. What counts as quality improvement and how this should be measured is a topic of much debate. First, there are issues with the very concept itself. It is increasingly being suggested that ‘quality of care’ is an inherently normative, rather than a descriptive, concept [6] and is thus far from uncontroversial. Second, there is debate on what we can learn from concrete examples of quality improvement. Interventions, such as the creation of networks, might work to improve quality in a particular context when particular conditions apply. There is no guarantee, however, that such interventions work in every context. The uncritical transplantation of quality improvement interventions is thus always a risky undertaking. A recent article raised the provocative question ‘Does quality improvement improve quality?’ [7]. The point is that even when particular interventions (for this paper, we will consider creation of networks) are suggested to be beneficial, they need to be justified in the particular context in which they will be implemented.

We believe the ethical obligations of health care networks are under discussed compared to other issues relating to such networks. Nevertheless, there is an urgent need to discuss such obligations from an organizational perspective. The difference between health care networks and individual health care institutions or collaborations of health care institutions is not merely size. Rather, health care networks are often amorphous and complex. They can be governed in a vast number of ways, assume any kind of form and involve a complex set of interactions [8]. While this increases the collective responsibility of the network, it makes it more difficult to locate and distribute individual responsibility and accountability [9]. How will fair access to those networks be guaranteed and by whom? How will patients following a care trajectory move throughout such a network? How will their medical information be safely stored, shared and transferred within such a complex network? Such issues need to be high on the agenda. We will not address each of these issues in depth but will look more broadly at ethical obligations we can expect such health care networks to meet.

### Business ethics perspective vs. patient perspective

When ethical obligations for networks are discussed, the issue is commonly approached from an organizational or a business ethical perspective [10–12]. This seemingly makes sense, as the question pertains to organizations such as health care networks and how they should be organized. Concrete discussions then include how legal and moral responsibility should be distributed within the broad network.

‘Business ethics’ can be understood as being ‘the study of the ethical dimensions of productive organizations and commercial activities’ [13]. It is clear that business ethics is not a single approach but rather a broad concept that can refer to a number of different approaches. Well-known business approaches include the shareholder approach, the stakeholder approach, [12] the corporate social responsibility approach [14] and the integrative social contracts theory [15]. However, despite the different approaches, they seem to share the same basic assumption. When applied to health care organizations and health care networks, business ethics starts by conceiving such organizations or networks as businesses. What exactly constitutes a business is a complicated and much debated topic [16]. Depending on the particular business ethics approach, this network then has to meet its fiduciary duties towards shareholders, stakeholder and/or society.

We argue that the choice for such a business ethics perspective is far from neutral and may raise concerns. While we believe that conceiving of HCNs as businesses might be possible, we argue that it is not desirable to do so. Considering health networks to be business entities involves considering them to be engaged in commercial activities and to see patients as ‘consumers’ of that service. Such a business approach may ignore the relevant ethical differences between patients and consumers of commercial services. First, patients are particularly vulnerable and are often in need of medical care. Second, the ‘service’ provided by health care networks is ‘medical care’ or ‘health’, which is a basic and common good. Being healthy is normally not a luxury good but a basic good that allows one to fully participate in society [17]. Making health care a commercial, sellable, purchasable and/or marketable good might degrade it [18]. Third, we know from research that patients do not operate as equal or rational consumers in the health care market. Most patients in need of a medical service do not compare different health care organizations or networks with regards to the quality and costs of that service [19]. These issues make it risky to start from the assumption that health care organizations are businesses.

Additionally, there is a risk that taking a business ethics approach might devaluate the central role of patients in medical care by conceiving of them as one stakeholder alongside other stakeholders. This may allow the interests of patients to be outweighed by the interests of other stakeholders. However, the ethical obligation to care first and foremost for the welfare of the patient lies at the very heart of medicine. We believe it is thus more fruitful to take an ethics approach to health care networks that starts from patients and the ethical claims they can make on these networks.

### The patient and the four principles

There is near perfect consensus that an ethical commitment to patient welfare is central to the practice of medicine. The obligation to care first and foremost for patients constitutes perhaps the strongest ethical obligation for health care professionals and is enshrined in codes of medical ethics worldwide [20, 21]. Patients can thus make strong ethical claims on health care institutions and networks.

A common way of categorizing these ethical claims is by making use of the well-known and widely accepted principles of Beauchamp and Childress: in clinical care, patients can expect health care networks to meet ethical duties of autonomy, beneficence, nonmaleficence, and justice [22]. Although this framework is often applied to the patient–physician relationship and the ethics of clinical care, we believe that this obligation transfers from individual health care professionals to HCNs. Whereas in clinical care, health care professionals have ethical duties towards individual patients, in HCNs, these duties take the form of more general ‘duties to design’ [23]. This involves obligations to design the network so that the interests of *all* patients (both actual and potential) within the network are protected.

### HCNs’ ethical duties towards patients

#### Justice and access to the network

It is widely acknowledged that all patients have an ethical claim to just and equitable access to health care [24]. The issue of justice is particularly important for HCNs, as they have the potential to significantly impact how health care is organized and delivered within a health care system. This is the case when they cover a larger geographical area or hold a de facto monopoly on the treatment of particular pathologies or the provision of particular services in a certain area. In these cases, the networks, rather than individual health care organizations, become the primary entity through which health care resources are distributed. The just, fair and equitable access to these networks is thus of crucial importance not only for the justifiability of the networks as such but also for the entire health care system in general.

Taking the perspective of the patient in need of medical care, there seems to be a prima facie right to access regardless of how or in what way one enters the network. If access is denied or limited, this should occur on justifiable grounds that are transparent, known to the patient and understandable from the patient’s perspective.

Naturally, much debate exists on what counts as ‘justifiable grounds’ for regulating access to health care in general or to HCNs in particular [25]. It is an undeniable truth that the resources of HCNs are limited and that an acceptable principle of distributive justice for allocating those limited resources must be chosen. Various possible distributive justice principles exists, and following Cookson and Dolan, these can be categorized as principles based on (medical) need, maximizing principles and egalitarian principles [26]. Beauchamp and Childress use six material principles of justice: (1) an equal share for each; (2) distribution according to need; (3) distribution according to effort; (4) distribution according to contribution; (5) distribution according to merit; and (6) distribution according to free-market exchanges [22]. It is important to emphasize that health care must be distributed in some way and that an unequal distribution is not automatically an unfair or unjust distribution.

Without entering the complex debate on justice in health care, we would argue that, following Norman Daniels’ idea of ‘accountability for reasonableness’ [27], HCNs have at least an obligation to be transparent about the allocation principles they apply and can be held accountable for these principles. Seen from the patient perspective, patients, we argue, have a right to know how and according to what principles access to a network is regulated. Based on this knowledge, they should be able to make a reasonable prediction about their own access to the network. This is also important to guarantee informed and autonomous patient choice.

Furthermore, even when there is debate about what constitutes proper grounds for allocating care, there can be consensus concerning what counts as unjustified grounds for distribution/allocation. There is wide consensus that factors such as social standing or sexual orientation can be problematic grounds. HCNs have a continuing obligation to monitor how they distribute their resources, and they have a duty to revise or adapt this distribution should it fail to meet minimally recognized standards of fairness.

Many factors determine access to health care, two of which are socioeconomic status and geography. First, an enormous amount of research confirms that higher socioeconomic status is associated with better, easier and/or faster access to health care. In the US context, much of the inequality in health care is attributed to socioeconomic inequality [28]. The same situation applies to other countries. Research indicates that in places such as Norway and England, higher socioeconomic status is associated with shorter waiting times [29, 30]. As regards geography, the decision by an HCN to offer a particular service at a single location might advantage those with the physical capacity and financial means to travel to this location. A literature review showed, for example, how travel burden can be a barrier for both the diagnosis and treatment of cancer [31]. Such knowledge should influence the organization of HCNs. For example, in Belgium, the government is planning to create 25 regional hospital networks, and every hospital (both private and public) will have to become part of one of these networks (based on their geographical location). The governmental policy stipulates that some basic medical services should be provided at every individual hospital of that network, whereas other medical services, such as maternity care, should be offered in some of the network’s hospitals but not all. Care must be taken that such a policy does not unduly interfere with patients’ right to autonomous choice of healthcare provider and, importantly, does not unfairly benefit those living close to the network hospitals offering a particular care. Care must be taken in organizing the provision of particular medical services within that network so that all possible patients within the network’s coverage have access.

#### Nonmaleficence and safety

A key ethical principle within medicine is the principle of *nonmaleficence*. This principle has often been linked to the well-known ‘first do no harm’ principle. However, as has been remarked by Beauchamp and Childress in their discussion of this principle, the concept of harm remains open to debate. A distinction is sometimes made between harming (understood as a setback of one’s interests) and wronging (understood as the violation of one’s legal or moral rights) [22]. One can, according to this view, thus be wronged without being harmed. For example, if a patient’s private medical data are shared with third parties who do not have justified access to these data, this patient is wronged, even if she was unaware of this sharing and suffered no setback of interests as a result. To include such cases, we take the nonmaleficence principle to be broader than the ‘first-do-no-harm’ principle and to also include the duty not to *wrong* patients.

Applied to HCNs, patients can make the reasonable ethical claim that receiving medical care within the network is not directly or indirectly harmful or wrongful. As regards harm, patients may be harmed in various ways, one of which is the direct physical harm that can occur when a patient is physically transferred. Some HCNs may cover large geographical areas, which might involve lengthy transfers for some patients. By taking the patient perspective, we believe it is clear that such risky transfers should always be justified in terms of the expected benefit *for the patient*. It is important that in a field of medicine with increasing attention to economic efficiency, rationing, cost-containment, and stakeholders, it is the patient and her safety that form the primary basis for ethical justifiability.

The ethical claims of patients to not be harmed extend, of course, beyond the transfer within a network. Even when patients are not transferred themselves, they might still be harmed and/or wronged, for example, when a patient’s health data are unjustly shared, thereby causing a breach of privacy [32]. Studies indicate that in the US, the exchange of health care information both within and between hospitals grew substantially between 2008 and 2012 [33]. In a time in which genetics is rapidly becoming increasingly important in medical care, a patient’s health data might also contain genetic data, which provide information not only about just the patient but also about his or her relatives who might not have voluntarily provided their data.

This issue of sharing patient information is particularly pressing for HCNs. Such networks have multiple partners, and sharing of information between these partners might be necessary, for example, to guarantee proper continuity of care or to improve quality of care. There is a tendency towards the creation of large shared data sets, such as the US Oncology Precision Network (OPeN), which contains clinical, genomic, pharmacological and treatment response data from oncology patients in 79 hospitals. Although the goal of such networks is to improve quality of care, there are reasons, we argue, to remain careful. One reason is that due to the often complex structure of HCNs, responsibility and accountability easily become diffused [9, 34]. We therefore believe that HCNs need strong and centralized agreements on how medical data are handled within the network, and power should be given to the patient who might not be aware of the ways their data are or can be used. Ethical concern has, for example, been raised about the possibility of buying and selling available health data [35].

The ethical duty to protect patient privacy accords with broader legal tendencies. Internationally, there is a clear drive towards more stringent data protection and putting people in charge of their data. An excellent example is the recent European General Data Protection Regulation (GDPR) [36], which aims to put people in charge of their own data, including medical data. In this sense, adequate protection of medical data is not only an ethical requirement for HCNs but also a legal one.

#### Beneficence and continuity of care

Patients can also make a legitimate claim that HCNs are organized to be maximally beneficent. First, this means that HCNs must be justified in terms of the degree to which they provide or improve quality of care. HCNs are often believed to improve quality, for example, due to the possibility of providing smooth and integrated care in a setting in which medical professionals from various disciplines work together [37]. Another advantage of HCNs in terms of quality might be that, within networks, particular medical interventions are or can be grouped together in one or more member institutions, which then results in a larger volume of these interventions for these members. Real life examples exist. In 2015, three US hospital systems took a volume pledge in which they pledged not to have certain specialized surgical procedures performed in hospitals where relatively few of those procedures were performed [38]. The aim was to promote the HCN’s outcomes, as empirical studies suggest that high volume is associated with better outcomes for certain treatments or surgical procedures [39] (although the magnitude of the association is sometimes questioned [40]).

Of course, although the general principle of increased quality may be valid for HCNs, this has to be shown to improve health care outcomes for each individual context. Additionally, the allocation of particular interventions to a particular HCN member must be justified by reference to *patient benefit.* Care must also be taken that the centralization of care in a particular network member does not result in a net loss of justice or patient autonomy. For example, in 2011, the US Carolinas HealthCare System Levine Cancer Institute covered 38 hospitals, and all patients had to travel to a central hospital for specialized cancer care. Such an organization is at risk of not meeting their ethical obligations of fairness and autonomy. More recently, the HCN switched its strategy with the creation of more than 20 decentralized centres and clinics [2].

Second, patients entering an HCN will follow a particular care *trajectory*, which might involve particular members of the HCN. The organization of this trajectory is largely irrelevant from the patient’s perspective. Patients not only have a right to quality care but also have an additional ethical right to smooth and high quality care *throughout their entire care trajectory*. Networks should thus be designed to maximally guarantee continuity of care. This involves three dimensions [41].

First, there should be informational continuity. HCNs have the obligation to ensure that relevant patient information is transferred safely and effectively for the benefit of the patient. The HCN must strike the right balance between sharing too much (thereby potentially harming the patient and breaching the duty of nonmaleficence) and sharing too little (thereby failing to maximally benefit the patient and breaching the duty of beneficence).

Second, HCNs should guarantee management continuity. There should be, within the network, a shared approach to the management of a particular health condition. Patients may suffer harm if they are transferred within a network only to be confronted with a different approach each time. It has been argued that, according to interviewed cancer specialists, one of the most significant barriers to high quality cancer care, for example, is ‘unnecessary variation in cancer care because of lack of standardization’ [42]. This would also be a breach of the ethical principle of nonmaleficence.

Third, there should be relational continuity. Physician–patient relationships remain the cornerstone of modern medicine. Research shows that trust in one’s primary physician is a reliable predictor of adherence to a treatment or medical regime [43]. When patients or their care are transferred within the HCN, so should the therapeutic relation and corresponding responsibility be. Without relational continuity, there is a risk of ‘collusion of anonymity’, in which ‘the patient is passed from one specialist to another with nobody taking responsibility for the whole person’ [44]. The trend towards HCNs carries with it the risk that patients are transferred without transfer of relational continuity. Again, due to the complex nature of HCNs, attribution and transfer of responsibility might be an issue. This is a classic case of a ‘problem of many hands’, in which collective undesirable effects occur without the possibility of holding any one individually responsible [9]. As such, HCNs need central arrangements regarding the distribution of responsibility and accountability so that at all times, someone is responsible and/or accountable and, perhaps more importantly, that someone also *feels* responsible. From the perspective of the patient, it is primarily the latter that is particularly important. As an example, one could consider the use of patient navigators in the US who help individual patients navigate the often complex US health care system [45]. Such a designated patient navigator could be justified by reference to the ethical principle of beneficence but has likewise been argued to help promote justice and equality [46].

#### Patient autonomy

Following the ethical principle of autonomy, HCNs have an ethical obligation to respect and actively promote patient autonomy. This principle may include the right to maximally make autonomous choices in one’s care trajectory and the right to choose one’s preferred health care professional or provider. The latter is, besides being a moral right, often also a recognized legal right in most jurisdictions.

However, this ethical duty to promote autonomy might in some situations seemingly conflict with HCNs’ ethical duty of beneficence. For example, an HCN might choose to allocate a certain health care service to one network member, thereby transferring all patients in need of this service to this member institution. From the perspective of beneficence, grouping all patients within a single institution might be justified. However, if networks are designed so that particular patients (or their data) are *automatically* transferred to particular institutions or patients are *automatically* put onto a particular care pathway, this could violate the ethical principle of patient autonomy, as patients are then unable to choose the institution they want to be treated in and the way in which they are treated. Earlier in the paper, we provided an example of a 38-hospital network in which all cancer patients had to travel to a central cancer centre to receive specialized cancer care [2].

One potential way out of this conflict is to use so-called clinical *nudges* [47, 48]. In principle, nudges are a form of choice-architecture in which, for example, particular policies are enacted that nudge patients towards a particular behaviour or choice, without overriding their right to free choice. Recently, there was a published plea for the creation of so-called ‘nudge units’ in health care institutions, which ‘systematically develop and test approaches using nudges to improve health care delivery’ [49]. In 2016, Penn Medicine started the Penn Medicine Nudge Unit, which devotes itself to testing such potential nudges.

Many examples of nudges in health care exist. A well-known and empirically tested example of a successful nudge is the use of default options [50]. In the context of HCNs, one could imagine a network in which patients with a particular pathology or in need of a particular medical service are transferred to a designated network member, but patients have the option to instead choose their own institution, should they prefer to do so. Such nudges allow for an HCN to become maximally efficient while protecting patient autonomy. If, for particular medical interventions, patients are referred to the network member with the largest volume for that intervention by default, this could increase quality of care and provide these patients with the best chance for the best outcome.

It has been argued that such nudges are ethically distinct from purely paternalistic interventions [48]. First, there is often no nudge-free way to design an HCN. Every health care network needs to be designed in some way, and this will inevitably influence patients’ choices in some way. The question is thus not whether nudges should be employed but rather the direction in which nudges should point. Second, even if certain choices might be facilitated, nudges always allow for free choice. There is no automatic overriding of choices patients make. Third, in some cases, nudges might actually help align patients’ choices with their own underlying values. For example, it is well established that patients often choose their health care provider because they are located near to it or because a particular provider has been recommended to them by their family or GP [19], rather than on medical or value-based grounds. One could design one’s network so that patients are more likely to end up with caregivers that provide the best possible care that aligns with their own particular value. This is distinct from hard paternalism, in which patient choices are overridden based on the caregivers’ values.

Many commentators remain sceptical about and doubt the justifiability of clinical nudges in some health care contexts[51–53]. Indeed, depending on how strong the nudge is, it always runs the risk of becoming a paternalistic intervention. For example, in the hypothetical case mentioned above, in which there is a default choice but patients are allowed to override the default option, patients may perhaps not be aware of the option to override. Others might be overly fearful of the consequences of overriding the default option. The mere presence of a possibility to override does not suffice for guaranteeing autonomous choice.

However, while we acknowledge that such critiques of nudges remain valid, we believe that they do not discredit the entire undertaking of nudges. As with the other principles discussed in this paper, we believe that the validity of nudges as a general principle does not justify the use of every kind of nudge in every kind of context. We are, instead, arguing that in the organization of networks, attention should be given to the relation between the way the network is designed and the kinds of patient decisions it incentivizes.

## Conclusions

Health care networks are well underway to assuming a more central role in tomorrow’s organized health care. A significant amount of research has been devoted to these networks. Nevertheless, their ethical aspects remain little discussed but are nevertheless pressing. Even when HCNs are shown to be beneficial, they still carry with them significant ethical risk. Due to their often amorphous and complex structure, individual responsibility may become highly diffuse, even up to a point where it is unclear who is responsible or accountable for what. In regard to issues as patient care and the safe storage and transfer of patient data, such a lack of individual responsibility may be problematic.

We therefore believe that it is high time to discuss ethical obligations we can expect HCNs to meet. We believe such a framework of ethical HCN obligations needs to start from the patient perspective rather than from the (as we have argued) more problematic business ethics perspective. For matters of clarity, we have categorized HCNs’ obligations towards patients using Beauchamp and Childress’ four principles of justice, nonmaleficence, beneficence and autonomy. For HCNs, this translates into duties to design the network so that it is responsive to patients’ medical needs and autonomous choices. More specifically, the principle of justice requires HCNs to provide just and equal access; nonmaleficence requires them to protect patients from harming *and* wronging; beneficence requires them to guarantee continuity of care; and autonomy requires them to allow patients to maximally make free choices in their care trajectory. Only when such obligations or duties are met in a particular context can the HCN be considered ethically justified.

## Abbreviation

HCN: Health care network
